# Identification of Two Novel Peanut Genotypes Resistant to Aflatoxin Production and Their SNP Markers Associated with Resistance

**DOI:** 10.3390/toxins12030156

**Published:** 2020-03-01

**Authors:** Bolun Yu, Huifang Jiang, Manish K. Pandey, Li Huang, Dongxin Huai, Xiaojing Zhou, Yanping Kang, Rajeev K. Varshney, Hari K. Sudini, Xiaoping Ren, Huaiyong Luo, Nian Liu, Weigang Chen, Jianbin Guo, Weitao Li, Yingbin Ding, Yifei Jiang, Yong Lei, Boshou Liao

**Affiliations:** 1 Key Laboratory of Biology and Genetic Improvement of Oil Crops, Ministry of Agriculture, Oil Crop Research Institute (OCRI) of Chinese Academy of Agricultural Sciences (CAAS), Wuhan 430062, China; yubolun@caas.cn (B.Y.); peanutlab@oilcrops.cn (H.J.); huangli01@caas.cn (L.H.); dxhuai@caas.cn (D.H.); zhouxiaojing@caas.cn (X.Z.); kangyanping@caas.cn (Y.K.); renxiaoping@caas.cn (X.R.); huaiyongluo@caas.cn (H.L.); lnian0531@caas.cn (N.L.); wgchen@caas.cn (W.C.); guojianbin@caas.cn (J.G.); 82101171080@caas.cn (W.L.); dingyingbing@caas.cn (Y.D.); jiangyifei@caas.cn (Y.J.); leiyong@caas.cn (Y.L.); 2 International Crops Research Institute of the Semi-Arid Tropics (ICRISAT), Hyderabad 502324, India; m.pandey@cgiar.org (M.K.P.); r.k.varshney@cgiar.org (R.K.V.); h.sudini@cgiar.org (H.K.S.)

**Keywords:** aflatoxin resistance, peanut, genome-wide association study (GWAS), mini-mini core collection, restriction site associated DNA sequencing (RAD-Seq)

## Abstract

Aflatoxin B_1_ (AFB_1_) and aflatoxin B_2_ (AFB_2_) are the most common aflatoxins produced by *Aspergillus flavus* in peanuts, with high carcinogenicity and teratogenicity. Identification of DNA markers associated with resistance to aflatoxin production is likely to offer breeders efficient tools to develop resistant cultivars through molecular breeding. In this study, seeds of 99 accessions of a Chinese peanut mini-mini core collection were investigated for their reaction to aflatoxin production by a laboratory kernel inoculation assay. Two resistant accessions (Zh.h0551 and Zh.h2150) were identified, with their aflatoxin content being 8.11%–18.90% of the susceptible control. The 99 peanut accessions were also genotyped by restriction site-associated DNA sequencing (RAD-Seq) for a genome-wide association study (GWAS). A total of 60 SNP (single nucleotide polymorphism) markers associated with aflatoxin production were detected, and they explained 16.87%–31.70% of phenotypic variation (PVE), with SNP02686 and SNP19994 possessing 31.70% and 28.91% PVE, respectively. Aflatoxin contents of accessions with “AG” (existed in Zh.h0551 and Zh.h2150) and “GG” genotypes of either SNP19994 or SNP02686 were significantly lower than that of “AA” genotypes in the mean value of a three-year assay. The resistant accessions and molecular markers identified in this study are likely to be helpful for deployment in aflatoxin resistance breeding in peanuts.

## 1. Introduction

As an important source of edible oil and protein for humans and livestock, the peanut (*Arachis hypogaea L*.) is widely grown in more than 100 countries, with China, India, and the United States being the largest producers [1]. Cultivated peanuts (*A. hypogaea*, AABB, 2n = 4x = 40) are believed to be derived from the diploid wild species *A. duranensis* (AA genome) and *A. ipaensis* (BB genome), originating in South America through heterologous hybridization and long-term domestication [2]. Peanuts are widely planted in China as an important oil crop. In recent years, China has witnessed continuous enhanced peanut productivity, which reached 17.33 million tons in 2018 [3]. The peanut has become the largest oilseed crop in terms of annual production for more than one decade; however, peanut farmers and industries in China, as well as in many developing countries, are facing serious challenges of an increased risk of aflatoxin contamination.

Aflatoxin B1, B2, G1, and G2 (AFB_1_, AFB_2_, AFG_1_, and AFG_2_) are the most toxic and carcinogenic naturally occurring mycotoxins produced by *Aspergillus* in peanuts. *Aspergillus flavus* is able to produce AFB_1_ and AFB_2_, while *Aspergillus parasiticus* can produce all four aflatoxins [4,5,6]. Restrictions on levels of aflatoxins in agricultural and food products have been set up in many countries in the world. China and the U.S. have set limits on levels of aflatoxin at 20 μg/g in food and feed, while the European Union has imposed stringent regulations on levels of aflatoxin at 2–4 μg/g [7]. Aflatoxin contamination can directly affect food safety, international trade, and market competitiveness of peanut products, resulting in enormous economic losses. Various measures, such as bio-control agents, good agricultural practices, and genetic improvement in host plants have been used to prevent and control aflatoxin contamination in peanuts [8,9,10,11]. It is well-known that risk of aflatoxin contamination can be effectively controlled by planting resistant peanut varieties combined with necessary crop management [12].

However, efforts at breeding for aflatoxin resistance in peanuts are highly limited by many factors, such as complex interactions among the peanut varieties, *Aspergillus* fungal strains, and environmental factors. Lack of desirable parental genotypes with reliable and high-level resistance has been a common constraint in peanut breeding programs. Besides, a lack of rapid and accurate methods for phenotypic identification of aflatoxin contamination is also a common technical drawback worldwide. Therefore, more precise identification of peanut materials resistant to aflatoxin contamination and development of molecular markers applicable for marker-assisted selection (MAS) in breeding are of great significance to reduce the risk of aflatoxin.

According to the flowering and branching characteristics, peanut germplasms have been divided into six botanical varieties or types. Peanut germplasm accessions and breeding lines, including GFA-1, GFA-2, AR-3, AR-4, Yueyou 9, and Zhonghua 6 were identified to be resistant to aflatoxins [13,14]. However, the improved peanut varieties with aflatoxin resistance that are available have only been used in relatively limited regions, due to their relatively low yield or other undesirable agronomic traits. In order to develop better aflatoxin-resistant peanut varieties, more systematic identification is needed to screen germplasms for resistance. Although over 8000 peanut germplasm accessions have been assembled in China, it is difficult to screen all of them for complex traits like aflatoxin resistance, which can only be accessed using complicated and dear cost method. Under such circumstances, phenotyping for aflatoxin resistance in the manageable peanut germplasm set, like the Chinese mini-mini core collection consisting of 99 diverse accessions, would be feasible [15].

The molecular mechanisms of the biosynthesis of aflatoxins have been well-investigated. The initial stage of aflatoxin biosynthesis is similar to that of fatty acid biosynthesis, where acetyl CoA acts as the start unit, and malonate monoacyl CoA acts as the extension unit to form aflatoxin’s polyketone skeleton, catalyzed by polyketide synthase (PksA) [16,17]. More than 18 enzymes, such as Nor-1 (oxidoreductase), AvnA (monooxygenase), AdhA (dehydrogenase), FAD (Flavin adenine dinucleotide)-containing monooxygenase, EstA (esterase), and VerB (versicolorin B desaturase) have been identified to be involved in the biosynthesis of aflatoxins [18]. Advances in plant proteomics and fungal genomics partly reveal the resistance mechanisms of aflatoxin contamination in host plants [19]. Three types of plant factors that may influence fungal growth and aflatoxin contamination have been involved in the processes of resistance mechanisms: The first type is seed proteins that act as fungal cell wall degrading enzymes; the second type is proteins or secondary metabolites from the host seed that could directly influence fungal growth and/or aflatoxin synthesis; and the third type is plant stress responsive proteins that are synthesized by the host in large amounts after infection by *A. flavus* [20]. Research on these resistance mechanisms may promote genetic improvement of aflatoxin contamination resistance in peanuts.

Advances in next-generation sequencing (NGS) have made genome-scale population genetic studies more straightforward and economical [12,21,22,23,24,25]. Restriction-site-associated DNA sequencing (RAD-Seq) is a fractional genome sequencing strategy that can identify large numbers of genetic variations, such as single nucleotide polymorphism (SNP), through sequencing genomes digested by restriction nuclease [26]. In peanuts, SNP markers obtained by RAD-seq have been successfully used for genetic linkage map construction and bulk segregant analysis (BSA) [27,28,29]. Resistance to aflatoxin accumulation in peanuts is a complex trait affected by several environmental factors. Strong interaction has been detected between environment factors and the genotype for aflatoxin contamination [20]. In our previous study, four major and six minor QTLs (quantitative trait locus) were identified for aflatoxin resistance through genome-wide QTL analysis with a genetic linkage map constructed by SSR (simple sequence repeats) markers [30]. Due to the limitation of the genetic linkage map’s resolution, the confidence interval’s genetic distance of these major QTLs was >7 cM (centimorgan). It is difficult to identify related candidate genes by such a large confidence interval. However, the genome-wide association study (GWAS) is an effective trait-mapping approach for identifying candidate genes that underlie complex phenotypic traits, and it has been applied in identifying associated markers and candidate genes for several important agronomic traits in peanuts [15,19,31], but has not yet focused on aflatoxin resistance. Therefore, the present study was performed to conduct GWAS in 99 accessions of the Chinese peanut mini-mini core collection, using RAD-Seq-based, high-density genotyping and phenotyping data for AFB_1_ and AFB_2_ contents from three environments via inoculation with *A. flavus* under laboratory conditions. This study successfully identified SNP markers and candidate genes associated with aflatoxin content in peanut seeds, which may open up further opportunities in developing a genomic solution for aflatoxin production resistance in peanuts.

## 2. Results

### 2.1. Phenotypic Variation for AFB_1_ and AFB_2_ in Chinese Mini-Mini Core

**Figure 1:**
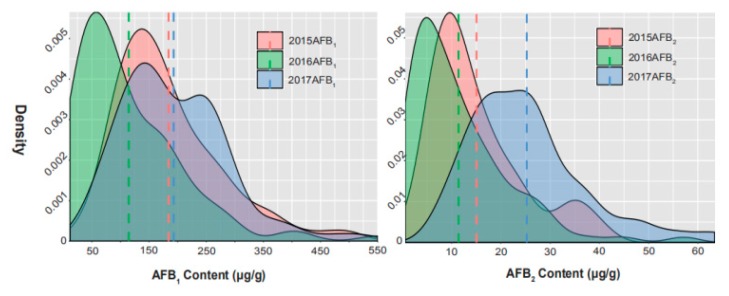
Phenotypic distribution of aflatoxin production in a three-year observation.

The phenotypic evaluation revealed a broad range of variations in aflatoxin production under the artificial inoculation assay among the 99 accessions of the Chinese peanut mini-mini core across 3 years (Figure 1, Table 1). The descriptive statistics of the phenotypic variations of three traits have been listed in Table 1. Across the three environments, the AFB_1_ and AFB_2_ content of the 99 accessions showed a continuous variation and approximated a normal distribution. AFB_1_ content ranged from 25.92 to 550.17 μg/g, with an average of 184.08 μg/g in 2015, from 11.69 to 505.01 μg/g, with an average of 114.45 μg/g in 2016 and from 26.08 to 526.21 μg/g, with an average of 193.01 μg/g in 2017. For AFB_2_, the content ranged from 0.98 to 41.60 μg/g, with an average of 15.06 μg/g in 2015, from 0.58 to 57.08 μg/g, with an average of 11.40 μg/g in 2016 and from 7.00 to 63.42 μg/g, with an average of 25.25 μg/g in 2017. The coefficient of variation (CV) of AFB_1_ in the environments of 2015, 2016, and 2017 were 0.51, 0.82, and 0.48, respectively, and for AFB_2_ it was 0.63, 0.88, and 0.47, respectively. Broad-sense heritability was estimated to be 0.57 for AFB_1_ content and 0.51 for AFB_2_ content (Table 2), indicating that both AFB_1_ and AFB_2_ content were partly controlled by genetic factors and affected by environmental factors. Variance analysis across the three trials also revealed that the genetic, environmental effects, and genotype × environment interaction significantly affected aflatoxin content.

Correlation coefficients between AFB_1_ and AFB_2_ were 0.88 (*p* < 0.001) in 2015, 0.99 (*p* < 0.001) in 2016, and 0.78 (*p* < 0.001) in the 2017 environment (Table 3). The significantly positive correlation between AFB_1_ and AFB_2_ suggests that resistance to AFB_1_ and AFB_2_ production in peanuts was controlled by the same genomic loci.

**Figure 2:**
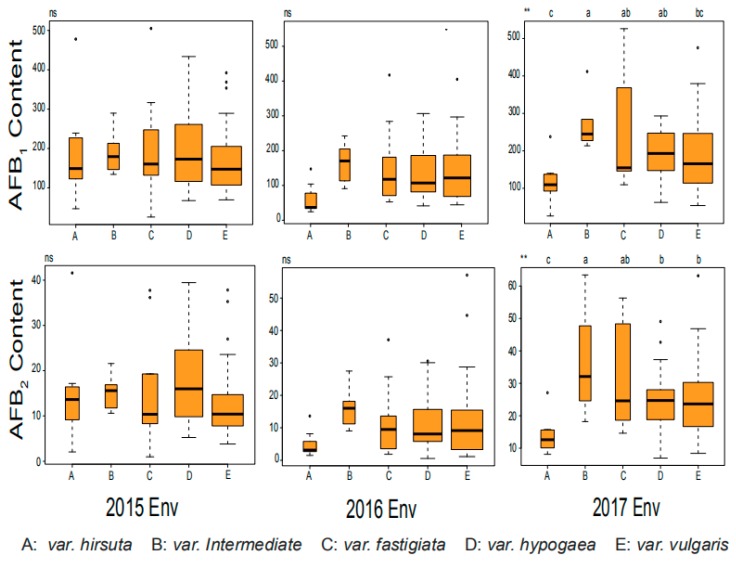
Box plot and multiple comparisons of aflatoxin production in five botanical types.

Two cultivated peanut accessions, Zh.h0551 and Zh.h2150, showed that extremely low content of both AFB_1_ and AFB_2_ and belonged to *var.hirsuta* and *var.vulgaris,* respectively (Table 4). The least significant difference (LSD) analysis was performed to compare the differences in AFB_1_ and AFB_2_ content among five botanical varieties involved, where the *var.intermediate* proved relatively higher than other varieties in both AFB_1_ and AFB_2_, and the *var.hirsuta* proved relatively lower than the other four varieties in both AFB_1_ and AFB_2_. In the 2017 environment, *var.hirsuta* proved to have a significantly lower level of AFB_1_ than the other three varieties (*var.intermediate*, *var.fastigiata,* and *var.hypogea*), and the var.intermediate proved to have a significantly higher AFB_1_ level than *var.hypogea* (Figure 2).

### 2.2. SNP Genotyping and Genetic Diversity

After filtering the SNPs with a call rate of <90% or with minor allele frequencies (MAFs) <5%, 36,058 polymorphic SNP markers were finally screened out and used to assess the population structure (Q), PCA analysis, relative kinship (K), and GWAS analysis. The filtered SNPs provided a 2.4 Gb genome-wide coverage with a mean distance of 118.44 Mb. Numbers of these SNPs ranged from 599 in A08, to 2387 in A03. The density of SNPs in each chromosome ranged from 49.93 kb/SNP in A10 to 87.85 kb/SNP in B01, with a mean density of 67.23 kb/SNP (Table 5).

### 2.3. Population Structure and Relative Kinship

**Figure 3:**
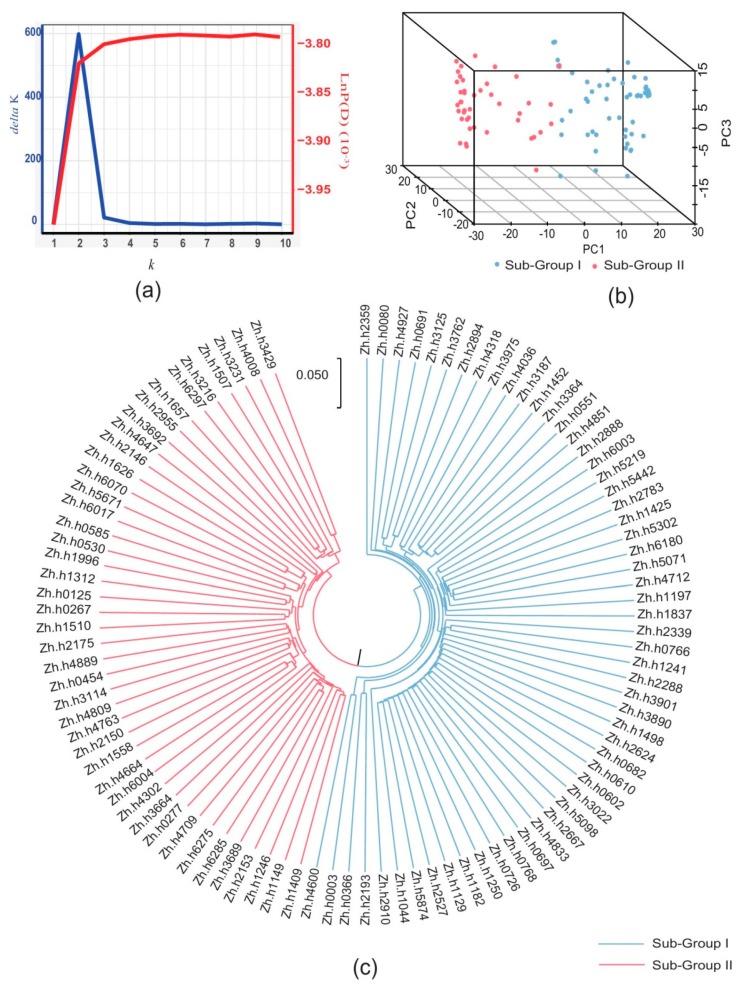
(**a**) Two different methods for determining the optimal value of K; (**b**) scatter plot of first three principal components of association panel; and (**c**) dendrogram of association panel based on genotype data.

The number of sub-populations of the 99 accessions was estimated based on the genotypic data. Bayesian clustering was performed for *k* = 1–10 with five repetitions, and significant changes were observed in both delta *k* value and LnP(D) value when *k* = 2 (Figure 3a). The UPGMA (unweighted pair group method using arithmetic average) phylogeny tree based on Nei’s genetic distances divided the 99 accessions into two subgroups (Figure 3c). The genotypic PCA three-dimensional plot also showed that the population was grouped into two major clusters, with few overlap regions (Figure 3b). The relative kinship between 99 genotypes was estimated based on the 36,058 SNP markers. A kinship coefficient less than 0.25 accounted for 98.60% of the association panel, indicating that the majority of the genotypes had a weak relationship with each other (Figure S1). The K matrix was visualized using a heat map, in which the two subgroups were clearly separated (Figure S2). Overall, these results suggested that the population of the Chinese mini-mini core collection could be divided into two subgroups: subgroup I and subgroup II. Subgroup I contained 55 accessions, of which 32 accessions (58.1%) belonged to *ssp.hypogaea*. Forty-five accessions were classified into Subgroup II, where 33 accessions (75%) belonged to *ssp.fastigiata* (Table S1).

### 2.4. Association Analysis and Candidate Genes

Five association analysis models, namely, the GLM (PCA), the GLM (Q), the MLM (Q + K), the MLM (PCA + K), and the MLM (K) model were compared and shown using a quantile quantile (QQ) plot to determine the most suitable model for association analysis. Based on the results of the QQ plot, the MLM (PCA + K) model was selected for GWAS (Figure S3).

**Figure 4:**
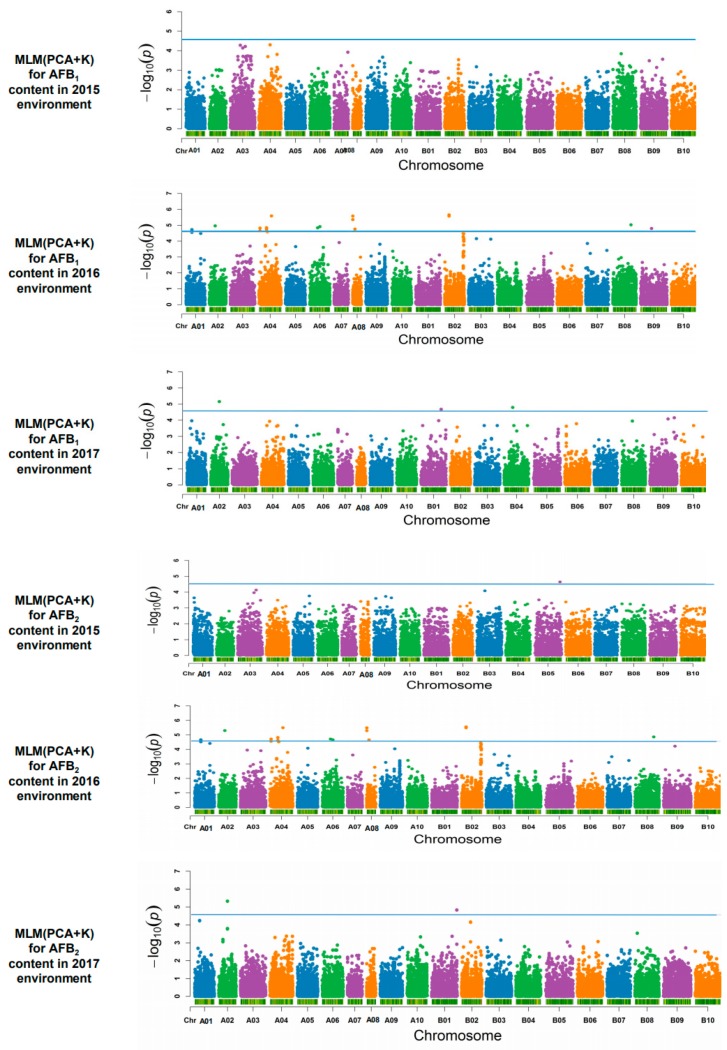
Genome-wide association for AFB_1_ and AFB_2_ content in peanut seeds.

A total of 60 SNP markers associated with AFB_1_ and AFB_2_ reached the corrected *p*-value threshold (−log^10^ (*p*) > 4.55). For AFB_1_, 32 significant SNP markers were detected in 10 chromosomes, with the PVE ranging from 16.87% to 28.83%. Among these markers, 28 associated SNP markers were detected in the 2016 environment, and four in the 2017 environment. Among these associated markers, SNP02428 in chromosome A02 which was detected in the 2016 environment showed the largest effect on AFB_1_ (PVE = 28.83%). For AFB_2_, 28 significant SNP markers were detected in nine chromosomes, with the PVE ranging from 22.27% to 31.70%. Among these associated markers, one marker was detected in the 2015 environment, 25 in the 2016 environment, and two in the 2017 environment. SNP02686 in chromosome A02, which was detected in the 2017 environment, had the largest effect on AFB_2_ content (PVE = 31.70%), and was also significantly associated with AFB_1_ (Table S2, Figure 4). Besides, as shown in Table 6, accessions with genotype “GG” or “GA” of SNP02686 accumulated significantly lower AFB_1_ and AFB_2_ than other “AA” genotypes in the mean value of three environments (Table 6). In addition, a total of 26 SNP markers were found to be associated with both AFB_1_ and AFB_2_ in the 2016 and 2017 environment (Table S2, Figure 4).

The whole genome was analyzed for the linkage disequilibrium (LD) block, where the size of the larger LD Block was mostly distributed around 200 kb (Figure S4). Based on this, a 100 kb candidate region on each side of the peak SNP marker associated with AFB_1_ and AFB_2_ was subsequently analyzed for identification of candidate genes. As a result, a total of 99 genes were identified in 15 candidate regions annotated in *A. duranensis* (A sub-genome) and *A. ipaensis* (B sub-genome). Aradu.WOPPM, located in the candidate region of the peak SNP marker SNP02686, was annotated as ATP-citrate lyase (ACLY), which is responsible for generating cytosolic acetyl-CoA and oxaloacetate. Acetyl-CoA is the substrate for the synthesis of aflatoxin. Genes coding WRKY transcript factors and proteins with a leucine-rich repeat domain were identified in the candidate region of the peak SNP marker SNP19994, which were reported as responsible for disease resistance in plants (Table S3).

## 3. Discussion

Occurrence of aflatoxin contamination in peanuts can have serious impacts on economies and human health worldwide. Development of peanut varieties with aflatoxin contamination resistance through genetic improvement is the most efficient solution to reduce risks of aflatoxin. This study is the first report to systematically identify associated molecular markers for aflatoxin accumulation resistance in peanuts. A total of 18 association peaks were identified for AFB_1_ and AFB_2_, with PVE ranging from 16.87% to 31.70%, including four peaks specific to AFB_1_, and two peaks specific to AFB_2_ (Table S2). According to annotation information of two ancestor wild *Arachis* species of cultivated peanuts (*A. duranensis* and *A. ipaensis*), 99 candidate genes were identified in 15 candidate genomic regions (Table S3).

Significant effects were detected in the genotype, environment, and genotype × environment interaction by a two-way ANOVA (Table 2). Several research reports indicated that the content of aflatoxin in peanut seeds was greatly affected by environmental factors during seed development. Fountain et al. (2017) pointed out that high temperature and drought stress had significant effects on the interaction between maize and *A. flavus* and the production of aflatoxin [32]. The same phenomenon was also observed in peanuts [33,34,35]. The reduced capacity of seeds to produce phytoalexins as the seed moisture content decreases during drought environment is believed to be an important factor for aflatoxin contamination [20]. In this study, as aflatoxin content is a trait that is highly affected by the environment, the phenotype of seeds harvested in the field varied greatly in different environments (Table 1, Table 2). Based on these reasons, no duplicate association SNP markers were found in different environments, neither for AFB_1_ nor for AFB_2_ (Table S2). However, except for 2015AFB_2_ vs. 2017AFB_2_, the Pearson correlations between the same phenotype in different environments were significant (Table S4). The selected set of peanut lines in our association panel showed stable performance for extreme phenotypes across three environments, with a 4- to 10-fold difference in aflatoxin content between resistant and susceptible genotypes (Table 4). These results indicated that screening aflatoxin-resistant materials through rapid identification of the mini core germplasm method is effective and that the identified resistant lines can be used for breeding aflatoxin contamination-resistant varieties.

In this study, only AFB_1_ and AFB_2_ were detected in peanut seeds, after artificial inoculation of AF2202 (*A. flavus*). The mean content of AFB_1_ in each sample was about ten folds of AFB_2_ (Table 1). The value of phenotypic correlations between AFB_1_ and AFB_2_ ranged from 0.78 to 0.99, with there being a significant level (*p* < 0.01) (Table 3). As a result, of the 19 SNP peaks detected in GWAS, 12 peaks were detected to be associated with both AFB_1_ and AFB_2_ (Table S2). It is noteworthy that both AFB_1_ and AFB_2_ are downstream products of versicolorin B (VB) in the aflatoxin metabolic pathway of *A. flavus* [36]. These results indicated that the mechanism of aflatoxin resistance in peanuts occurs mainly in the upstream metabolic pathway of VB production. Besides, in the AFB_1_ synthesis pathway, versicolorin A (VA) is the downstream product of VB, which contains the 2, 3 double bond in the dihydrobisfuran ring. This double bound can be oxidized in the host organism [18]. These studies could explain why the content of AFB_1_ is much higher than that of AFB_2_ in each sample of this study.

GWAS is considered to be an efficient genetic analysis method for complex traits. There are several elements that affect the precision of GWAS, such as population size, marker density, and statistical methods. In theory, an association population with large number accessions that encompasses the genetic diversity as much as possible is an optimal choice for association analysis. However, the operation of this experiment is relatively tedious, and systematic errors are easily introduced in seed sample selection, pre-treatment, artificial inoculation, and liquid chromatography measurements during the use of large populations. Hence, a mini core collection is a useful strategy [15]. The population structure and relative kinship were calculated by the genotype of association panel, and then used in five GWAS statistical methods to control false-positive results. Similar to other studies using peanuts [37,38], the 99 accessions of the Chinese mini-mini core collection in this study were classified into two subgroups (Figure 3).

Breeding for peanut varieties for resistance to aflatoxin contamination is the most effective approach for reducing hazards by aflatoxin. In a previous study, major QTLs for aflatoxin contamination resistance in peanuts were identified in chromosome A07 and B06 by linkage analysis with a genetic linkage map developed by a SSR marker [30]. However, because of the limited density of SSR markers in the genetic map, the confidence intervals of these QTLs are very large (7–16 cM). It is difficult to identify related resistance candidate genes by such a large confidence interval. In this study, a total of 18 association peaks distributed in 11 chromosomes were identified as associated with aflatoxin content in peanut seeds across multiple environments, 12 of which were associated with both AFB_1_ and AFB_2_, four were associated with AFB_1_, and two were associated with AFB_2_ (Table S2). These results imply that the resistances to AFB_1_ and AFB_2_ in peanut seeds largely share the same mechanism controlled by multiple genes. In plants, proteins with a leucine-rich repeat (LRR) domain have been confirmed to be involved in processes of plant disease resistance. These proteins work as the first point of pathogen defense, where the innate immune response is initiated through the sensing of pathogen-associated molecular patterns (PAMPs). Two genes encoding a LRR domain were identified in chromosome B01 in a 50 kb candidate genomic region of the SNP marker SNP19994; besides, a gene encoding WRKY transcription factor was also identified in this genomic region. The SNP marker SNP02686, which has shown the largest PVE for AFB_2_, was also associated with AFB_1_ in the 2017 environment. The Aradu.WAPPM gene was located 33.18 kb from SNP02686 in chromosome A02, which was predicated to encode ATP-citrate lyase (ACLY), responsible for generating cytosolic acetyl-CoA and oxaloacetate (Table S3). In *A. flavus*, acetyl-CoA is the key substrate of the aflatoxin biosynthesis pathway. Although there is no direct evidence that acetyl-CoA produced in peanuts is involved in the biosynthesis of aflatoxin in *A. flavus*, the findings of this study can provide ideas for further research. Peanut accessions with “AG” (contained in Zh.h0551 and Zh.h2150) and “GG” genotypes of either SNP19994 or SNP02686 possessed significantly lower aflatoxin content than that of the “AA” genotype. Further study of these markers may contribute to the development of aflatoxin production diagnostic molecular markers.

## 4. Conclusions

The present study reported the phenotyping of aflatoxin resistance in the Chinese peanut mini-mini core collection, followed by genotyping using the RAD-Seq approach and association mapping. Two peanut accessions (Zh.h0551 and Zh.h2150) were identified as resistant to aflatoxin contamination, with stable performance in multiple environments. The identified SNP markers for resistance to aflatoxin contamination along with the resistant materials would be helpful for peanut breeding for aflatoxin resistance.

## 5. Materials and Methods

### 5.1. Peanut Plant Materials

The 99 peanut accessions of the Chinese mini-mini core collection were planted in an experimental field (sandy soil) of the Oil Crops Research Institute of Chinese Academy of Agricultural Sciences (OCRI-CAAS) in Wuhan over three consecutive years (2015, 2016, and 2017), using a randomly complete block design with three replications. Each accession was planted in three rows with 10–15 plants in each row. The distance between each row was 30 cm, and there was 10 cm between plants in each row. Field management followed the standard agricultural practices. Continuously meteorological data in the field (including air temperature, relative humidity, monthly precipitation, and soil temperature) were observed by the Dynamet Weather 2 Station (Table S5). Soil pH, soil organic matter (SOM) content, soil total nitrogen (STN) content, soil available phosphorous (SAP) content, and soil available potassium (SAK) in the field were collected before sowing (Table S6). The Soil pH was determined by pH meter (STARTER 3100M, OHAUS, Newark, NJ, USA); SOM content was determined using the Walkley–Black oxidation method [39]; STN content was measured using the Kjeldahl procedure (UDK140 Atomatic Steam Distilling Unit, VELP Scientifica, Milan, Italy) [40]; SAP content was determined using the molybdenum antimony colorimetric method, after digestion by NaHCO_3_ (0.5 mol/L) [40]; and SAK content was determined by the flame spectrometry method, after extraction with NH_4_OAc (1 mol/L) [40]. After harvesting, all peanuts were dried immediately and the moisture content of the seeds was controlled to 5%–8%. Healthy and mature peanut seeds were artificially selected for further analysis.

### 5.2. Phenotyping for Aflatoxin Production

The toxigenic *Aspergillus flavus* strain AF2202 was maintained in 20% glycerol at conditions of −80 °C in OCRI-CAAS, China. The *A. flavus* AF2202 strain spores from vial stocks were cultured on fresh potato dextrose agar medium in a 90 mm petri dish at 30 °C for 7 days. Conidia were collected from Petri dishes and then suspended in sterile water containing 0.05% Tween-80 (Sigma-Aldrich, Munich, Germany) in a flask. The spore suspension was then diluted to 2 × 10^6^ CFU/mL in 0.05% Tween solution. For each accession of association panel, about 80 healthy and mature seeds were selected and sterilized with 75% ethanol for 1 min, followed by three washes with sterile water. The sterilized peanut seeds were randomly and evenly assigned to four sterilized Petri dishes as three repeated inoculation groups and one blank control group. The inoculation groups were inoculated by applying 1 mL of the above conidial suspension in sterile Petri dishes. The control groups were treated by the same inoculation method, and 1 mL of 0.05% Tween-80 instead of 1 mL conidial suspension was applied. In order to ensure the consistency of water absorption in different accessions, the contact time between peanut seeds and liquid during sterilization and inoculation was strictly controlled at 13 min. After inoculation, all Petri dishes were cultured in an artificial climate incubator (RZH-500A, Huier, Hangzhou, China) for 7 days with 85% relative humidity, 30 °C air temperature, and a dark environment.

After being co-cultured with *A.flavus,* all peanut seeds were dried at 110 °C for 2 h, grounded to fine powder using a coffee blender, and then filtered by a 20-mesh sieve. About 10 g of peanut powder was transferred into a 500 mL erlenmeyer flask with stopper capacity, mixed with 50 mL methanol (analytically pure grade, Xilong, Shantou, China)/water (55:45 v/v) and 15 mL petroleum ether (analytically pure grade, Xilong, Shantou, China), and shaken at 200 rpm at room temperature for 30 min. The crude extraction was filtered through qualitative filter paper (medium speed, Whatman, General Electric Company, Boston, MA, USA) and then diluted 10 times by 55% methanol. Separation and quantitation of aflatoxins was performed using a HPLC instrument (Agilent Technologies 1200 series, Santa Clara, CA, USA) equipped with a HPLC C18 4.6 mm × 250 mm, 5 μm column (Agilent, Santa Clara, CA, USA). The column was maintained at 30 °C in the system column heater. The mobile phase was composed of a methanol/ water (45:55) mixture, where the flow rate was 0.7 mL/min. The injection volume was 10 μL, and the injection time was 25 min. Concentration of aflatoxins were determined by peak areas with regression equations:

(1) $$y_{1}=\frac{1.4906x_{1}-0.0524}{1000}$$

(2) $$y_{2}=\frac{0.5618x_{2}+0.0866}{1000}$$

(y_1_ for concentration of AFB_1_, x_1_ for peak area of AFB_1_, y_2_ for concentration of AFB_2_, and x_2_ for peak area of AFB_2_) based on standard curves. The standard curves were established by the afaltoxin standard solution (CRM46304, Sigma-Aldrich, Munich, Germany). The lowest detection limit was 0.003 μg/g for AFB_1_ and 0.001 μg/g for AFB_2_.

### 5.3. RAD-Seq and SNP Calling

The genomic DNA of the 99 accessions were isolated from the young leaves of a single plant using a QIAGEN DNAeasy Plant mini kit (Dusseldorf, Germany), and operated according to the product manual. A total of 150 ng of genomic DNA of each accession was digested by 5 U of SacI and MseI (Thermo Fisher, Waltham, MA, USA). The digest react condition was set at 37 °C for 6 h and then 65 °C for 90 min. For each sample, 10 pmol of SacI and MseI adaptors (Table S7) together with 1000 U of T4 ligase (New England Biolabs, Ipswich, MA, USA) were used for ligation reaction in a 50 μL reaction system at 16 °C overnight. The ligation products of each sample were amplified by PCR (Polymerase Chain Reaction) in a 50 μL reaction system with 4 pmol of two overhang primers (Table S8), 1rHF buffer, 3.5 mM Mgcl_2_, 0.4 mM dNTPs, and 0.5 U of iproof polymerase (Bio-Red, Berkeley, CA, USA). PCR amplification was performed as follows: 98 °C for 2 min, followed by 12 cycles at 98 °C for 30 s, 65 °C for 30 s, and 72 °C for 15 s, and a final extension at 72 °C for 5 min. These PCR products (300–500 bp) were extracted after agarose gel electrophoresis using a Gel Extraction Kit (Qiagen, Dusseldorf, Germany) and then quantified by Agilent 2100 Bioanalyzer (Agilent Technologies, Santa Clara, CA, USA). Finally, these products were submitted for sequencing on the Illumina Hiseq2000 platform (Illumina, San Diego, CA, USA).

After quality control and filtering, all reads were mapped on the reference genome (A-subgenome, *Arachis duranensis*; B-subgenome, *Arachis ipaensis*, https://peanutbase.org/home) [41]. SNPs were identified by GATK and SAMtools, and filtered with minor allele frequency (MAF) and integrity (genotyped rate) [42,43].

### 5.4. Population Structure, Relative Kinship, Phylogenetic Tree Construction, and Linkage Disequilibrium

Population structure (Q-matrix) was calculated using Structure 2.2 software [44]. The number of possible subgroups (*k*) was pre-defined as 1–10 to explore the population structure of the tested accessions. The maximum likelihood estimates were applied to achieve reliable subgroups (*k*) with each *k* being run five times. The optimum *k*-value was obtained by the methods described by Evanno et al. [45]. The relative kinship, phylogenetic trees, principal component analysis (PCA), and linkage disequilibrium (LD) estimate were performed using Tassel 5 software based on the SNP genotype of the 99 accessions [46].

### 5.5. GWAS for Aflatoxin Production in Peanut Seeds

GWAS was conducted using Tassel 5 software [46] with a general linear model (GLM) and mixed linear model (MLM). An adjusted Bonferroni method was used to correct the multiple tests of GWAS, in which the *p*-value threshold was at *p* = 2.77 × 10−5 (1/*n*, *n* was the number of markers for GWAS) [47]. The genomic region of 100 kb upstream or downstream from a peak SNP marker was used for candidate gene identification.

## Figures and Tables

**Table 1:** Phenotypic variations of AFB_1_ and AFB_2_ of the association panel.

Traits	Env ^c^	Range	Mean	SD ^d^	CV ^e^
AFB_1_ (μg/g) ^a^	2015	25.92–550.17	184.08	93.14	0.51
	2016	11.69–505.01	114.45	94.39	0.82
	2017	26.08–526.21	193.01	91.84	0.48
AFB_2_ (μg/g) ^b^	2015	0.98–41.60	15.06	9.42	0.63
	2016	0.58–57.08	11.40	9.99	0.88
	2017	7.00–63.42	25.25	11.88	0.47

^a^ aflatoxin B_1_ content; ^b^ aflatoxin B_2_ content; ^c^ environment; ^d^ standard deviation; ^e^ coefficient of variation.

**Table 2:** Analysis of variance for AFB_1_ and AFB_2_ in the association panel across three environments.

Traits	Source	DF ^c^	SS ^d^	MS ^e^	*F* Value	*p* Value	*б^2^* ^f^	*h^2^* *^g^*
AFB_1_^a^	Genotype	98	4106986	41908	9.18	<0.001	2667.69	0.57
	Environment	2	1077196	539598	117.97	<0.001	1756.56	
	Genotype × Environment	196	3508164	17899	3.92	<0.001	4444.76	
	Error	594	2711315	4565			4564.50	
AFB_2_^b^	Genotype	98	48863	499	5.96	<0.001	28.52	0.51
	Environment	2	30454	15227	182.19	<0.001	50.45	
	Genotype × Environment	196	47409	242	1.20	<0.001	52.77	
	Error	594	49644	84			83.58	

^a^ aflatoxin B_1_ content; ^b^ aflatoxin B_2_ content; ^c^ degree of freedom; ^d^ sum of squares; ^e^ mean square; ^f^ variance; ^g^ broad-sense heritability.

**Table 3:** Pearson correlation between AFB_1_ and AFB_2._

Env ^a^	Pearson Correlation between AFB_1_ and AFB_2_	*p*-Value
2015	0.88	<0.01
2016	0.99	<0.01
2017	0.78	<0.01

^a^ environment.

**Table 4:** Extreme peanut accessions with aflatoxin production resistance.

Traits	Group	Accession Number	Var Type	2015Env ^c^	2016Env	2017Env	Average
AFB_1_(μg/g) ^a^	Susceptible control	Zh.h4600	*var.vulgaris*	392.31	450.17	310.45	384.31
		Zh.h3231	*var.fastigiata*	247.43	409.57	368.59	341.86
	Low content	Zh.h0551	*var.hirsuta*	39.00	21.42	33.08	31.17
		Zh.h2150	*var.vulgaris*	49.74	20.71	31.10	33.85
AFB_2_(μg/g) ^b^	Susceptible control	Zh.h4600	*var.vulgaris*	37.83	45.08	25.55	36.15
		Zh.h3231	*var.fastigiata*	28.27	32.13	44.32	34.91
	Low content	Zh.h0551	*var.hirsuta*	8.33	5.19	6.32	6.61
		Zh.h2150	*var.vulgaris*	5.27	4.13	9.57	6.32

^a^ aflatoxin B_1_ content; ^b^ aflatoxin B_2_ content; ^c^ environment.

**Table 5:** The number and density of SNP markers detected across peanut chromosomes.

Chromosome	SNP Number	Marker Start Loci (Kb)	Marker End Loci (Mb)	Reference Length (Mb)	Density of Markers (kb/SNP)
A01	1856	1621.08	106.90	105.28	56.72
A02	1512	165.40	93.53	93.36	61.75
A03	2387	69.80	134.58	134.51	56.35
A04	2317	277.02	123.31	123.03	53.10
A05	2019	252.20	109.66	109.41	54.19
A06	1774	302.81	112.63	112.32	63.32
A07	1116	201.97	79.09	78.89	70.69
A08	599	519.89	49.09	48.57	81.09
A09	2125	276.41	120.36	120.08	56.51
A10	2184	156.37	109.21	109.05	49.93
B01	1560	136.00	137.19	137.05	87.85
B02	1635	137.78	108.93	108.79	66.54
B03	1967	101.11	135.70	135.60	68.94
B04	2000	176.67	133.52	133.35	66.67
B05	1757	3440.92	149.75	146.31	83.27
B06	1743	124.59	135.87	135.75	77.88
B07	1608	247.60	126.20	125.95	78.33
B08	1864	578.78	129.60	129.02	69.22
B09	2373	135.20	146.85	146.72	61.83
B10	1689	34.68	135.89	135.86	80.44

**Table 6:** Phenotypic effect of SNP02686 and SNP19994 in association panel.

SNP Marker	Genotype	*n*	AFB_1_	AFB_2_
SNP02686	AA	3	312.57 ± 21.93 ^a^	38.15 ± 1.59 ^a^
	GG	6	195.35 ± 37.99 ^b^	19.20 ± 7.24 ^b^
	GA	80	159.88 ± 62.86 ^b^	16.57 ± 6.54 ^b^
SNP19994	AA	9	223.56 ± 38.64 ^a^	25.37 ± 9.12 ^a^
	GG	3	160.75 ± 34.74 ^b^	16.62 ± 4.76 ^b^
	GA	79	145.08 ± 31.12 ^b^	14.25 ± 2.67 ^b^

*n*: number of genotypes.

## Supplementary Materials

The following are available online at https://www.mdpi.com/2072-6651/12/3/156/s1, Figure S1: Distribution of pairwise relative kinship estimates, Figure S2: Heat-map of pairwise relative kinship estimates Figure S3: QQ plot for AFB_1_ and AFB_2_, Figure S4: LD decay, Table S1: Information for the 99 accessions used in this study, Table S2: Significant markers detected for AFB_1_ and AFB_2_ content in peanut seed, Table S3: Candidate genes information, Table S4: Pearson correlation for AFB_1_ and AFB_2_. Table S5: Meteorological data of peanut cultivation. Table S6: Soil nutrient content in the experimental field. Table S7, Sequences of adapters used in ligation reaction. Table S8, Overhang primers for polymerase chain rection.
