# Supplementary material for: Identification of Two Novel Peanut Genotypes Resistant to Aflatoxin Production and Their SNP Markers Associated with Resistance
Source: Toxins (Basel). 2020 Mar 1;12(3):156. doi: 10.3390/toxins12030156 (PMC7150746; doi:10.3390/toxins12030156)
Supplement: Supplementary file 1 [file toxins-12-00156-s001.pdf]

Supplementary Materials: Identification of Two Novel Peanut Genotypes Resistant to Aflatoxin Production and Their SNP Markers Associated with Resistance

Bolun Yu, Huifang Jiang, Manish K. Pandey, Li Huang, Dongxin Huai, Xiaojing Zhou, Yanping Kang, Rajeev K. Varshney, Hari K. Sudini, Xiaoping Ren, Huaiyong Luo, Nian Liu, Weigang Chen, Jianbin Guo, Weitao Li, Yingbin Ding, Yifei Jiang, Yong Lei and Boshou Liao *

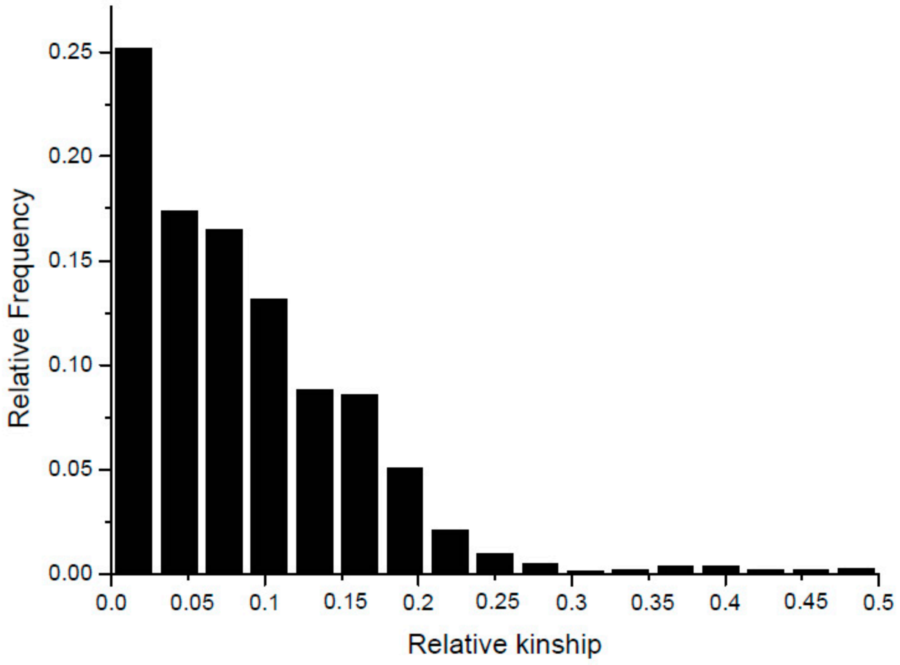

Figure S1. of pairwise relative kinship estimate.

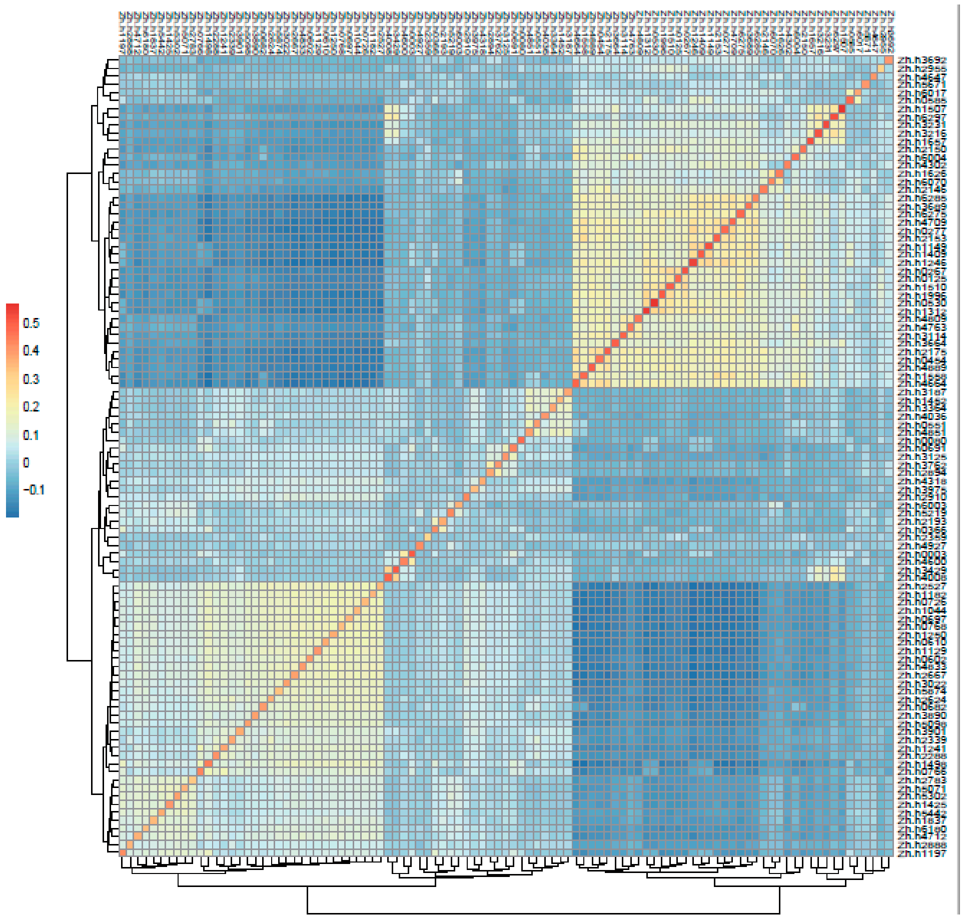

Figure S2. Heat-map of pairwise relative kinship estimates.

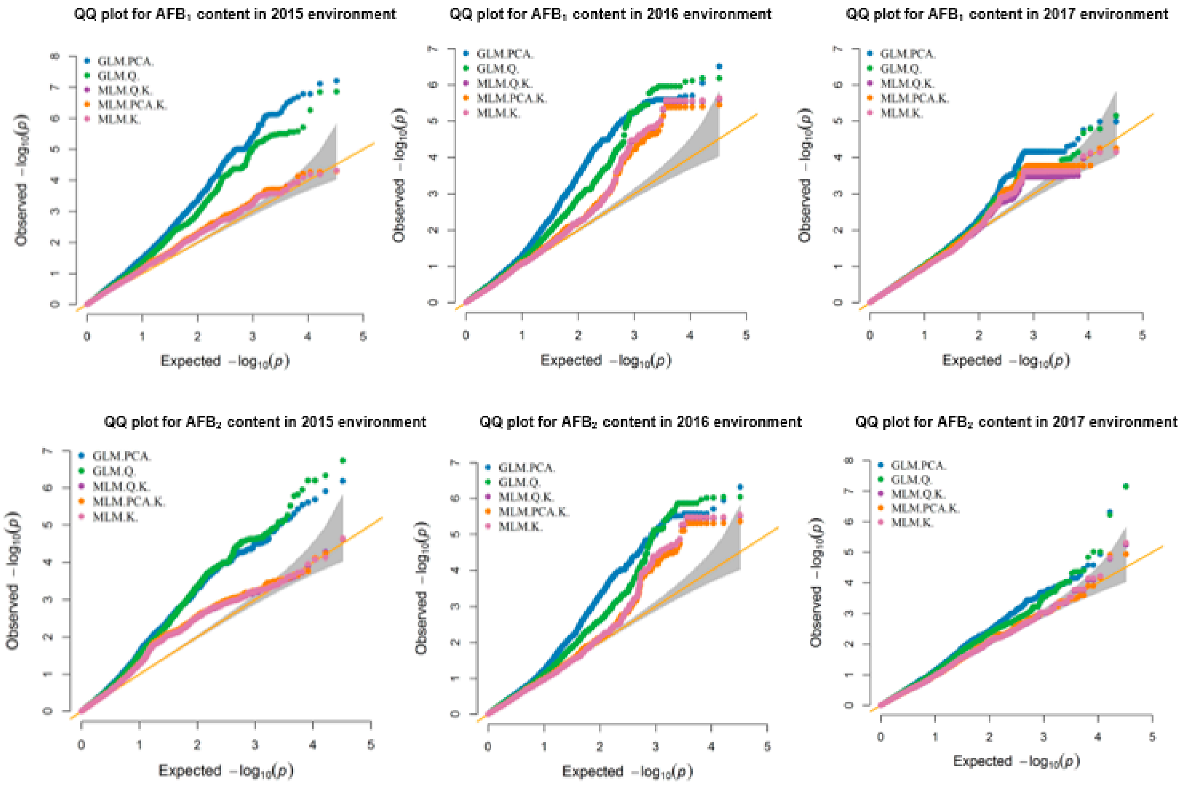

Figure S3. QQ plot for AFB1 and AFB2.

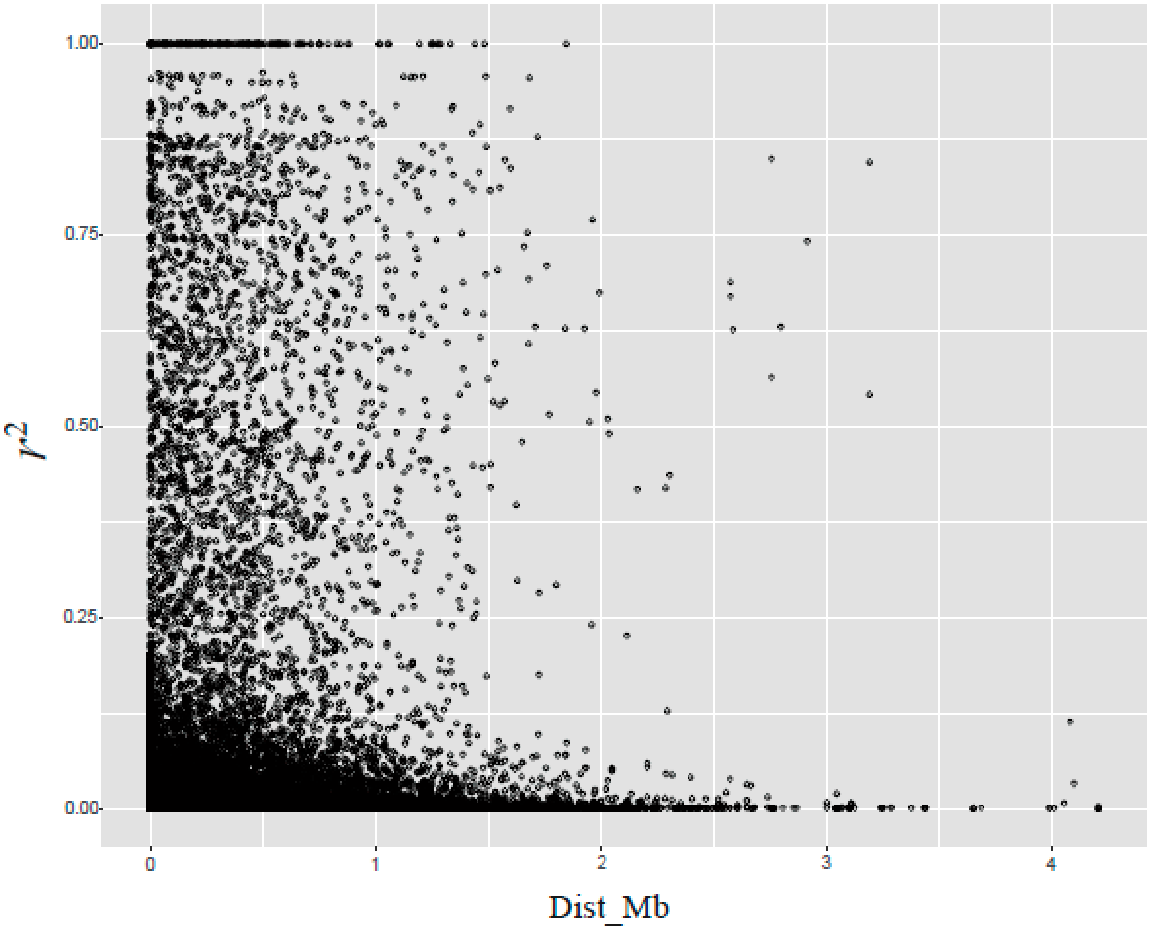

Figure S4. LDdecay.

Table S1. Information for the 99 accessions used in this study.

Accession	Var	Sub-Group
Zh.h1044	var. hypogaea	I
Zh.h5874	var. vulgaris	I
Zh.h2527	var. hypogaea	I
Zh.h1129	var. hypogaea	I
Zh.h1182	var. hypogaea	I
Zh.h1250	var. hypogaea	I
Zh.h0726	var. hypogaea	I
Zh.h0768	var. hypogaea	I
Zh.h0697	var. hypogaea	I
Zh.h4833	var. vulgaris	I
Zh.h2667	var. hypogaea	I
Zh.h5098	var. hypogaea	I
Zh.h3022	var. hypogaea	I
Zh.h0602	var. hirsuta	I
Zh.h0610	var. hirsuta	I
Zh.h0682	var. hypogaea	I
Zh.h2624	var. hypogaea	I
Zh.h1498	var. fastigiata	I
Zh.h3890	var. hypogaea	I
Zh.h3901	var. hypogaea	I

Zh.h2288	var. hirsuta	I
Zh.h1241	var. hypogaea	I
Zh.h0766	var. hypogaea	I
Zh.h2339	var. hirsuta	I
Zh.h2910	var. vulgaris	I
Zh.h1837	var. vulgaris	I
Zh.h1197	var. hypogaea	I
Zh.h4712	var. vulgaris	I
Zh.h5071	var. hypogaea	I
Zh.h6180	Intermediate	I
Zh.h5302	Intermediate	I
Zh.h1425	var. hypogaea	I
Zh.h2783	Intermediate	I
Zh.h5442	Intermediate	I
Zh.h5219	Intermediate	I
Zh.h6003	var. vulgaris	I
Zh.h2888	var. vulgaris	I
Zh.h4851	var. vulgaris	I
Zh.h0551	var. hirsuta	I
Zh.h3364	var. fastigiata	I
Zh.h1452	var. hypogaea	I
Zh.h3187	var. fastigiata	I
Zh.h4036	var. hypogaea	I
Zh.h3975	var. hypogaea	I
Zh.h4318	var. hypogaea	I
Zh.h2894	var. vulgaris	I
Zh.h3762	var. vulgaris	I
Zh.h3125	var. hypogaea	I
Zh.h0691	var. hypogaea	I
Zh.h4927	var. hypogaea	I
Zh.h0366	var. vulgaris	I
Zh.h2193	var. vulgaris	I
Zh.h0080	var. vulgaris	I
Zh.h4600	var. vulgaris	I
Zh.h0003	var. fastigiata	I
Zh.h2359	var. hirsuta	II
Zh.h1409	Intermediate	II
Zh.h1149	var. hypogaea	II
Zh.h1246	var. hypogaea	II
Zh.h2153	var. vulgaris	II
Zh.h3689	var. vulgaris	II
Zh.h6285	var. vulgaris	II
Zh.h6275	var. fastigiata	II
Zh.h4709	var. vulgaris	II
Zh.h0277	var. vulgaris	II
Zh.h3664	var. vulgaris	II
Zh.h4302	var. vulgaris	II
Zh.h6004	var. vulgaris	II
Zh.h4664	var. vulgaris	II
Zh.h1558	var. vulgaris	II
Zh.h2150	var. vulgaris	II
Zh.h4763	var. vulgaris	II

Zh.h4809	var. vulgaris	II
Zh.h3114	var. hypogaea	II
Zh.h0454	var. vulgaris	II
Zh.h4889	var. vulgaris	II
Zh.h2175	var. vulgaris	II
Zh.h1510	var. fastigiata	II
Zh.h0267	var. vulgaris	II
Zh.h0125	var. vulgaris	II
Zh.h1312	var. hypogaea	II
Zh.h1996	var. vulgaris	II
Zh.h0530	var. hirsuta	II
Zh.h0585	var. hirsuta	II
Zh.h6017	var. vulgaris	II
Zh.h5671	var. hypogaea	II
Zh.h6070	var. vulgaris	II
Zh.h1626	var. vulgaris	II
Zh.h2146	var. vulgaris	II
Zh.h4647	var. vulgaris	II
Zh.h3692	var. vulgaris	II
Zh.h2955	var. hypogaea	II
Zh.h1657	var. vulgaris	II
Zh.h6297	var. vulgaris	II
Zh.h3216	var. fastigiata	II
Zh.h1507	var. fastigiata	II
Zh.h3231	var. fastigiata	II
Zh.h4008	var. hypogaea	II
Zh.h3429	var. fastigiata	II

Table S2. Significant markers detected for AFB1 and AFB2 content in peanut seed.

Trait	Env	Marker	Chromosome	Position	*p*-Value	PVE
	2016	*SNP00538	A01	34209806	2.35E-05	25.85%
	2016	*_**SNP00539**_	A01	34209850	1.92E-05	26.41%
	2016	*SNP00541	A01	34209885	2.38E-05	25.75%
	2016	*SNP00542	A01	34209906	2.38E-05	25.79%
	2016	*SNP00543	A01	34209910	2.38E-05	25.79%
	2016	*_**SNP02428**_	A02	32402181	1.11E-05	28.83%
	2016	SNP05985	A04	5449323	2.26E-05	27.07%
	2016	*_**SNP05994**_	A04	5449452	1.52E-05	27.21%
	2016	*SNP06001	A04	5449524	1.52E-05	27.21%
	2016	*_**SNP06730**_	A04	41670901	1.43E-05	26.48%
AFB_1	2016	*SNP06731	A04	41670932	1.83E-05	26.22%
	2016	**SNP06816**	A04	47043012	2.63E-05	26.08%
	2016	*_**SNP07247**_	A04	69306120	2.63E-06	25.89%
	2016	*_**SNP11095**_	A06	44574792	1.46E-05	27.15%
	2016	*SNP11099	A06	44574866	1.46E-05	27.15%
	2016	*_**SNP11310**_	A06	57503242	1.23E-05	28.37%
	2016	*SNP13362	A08	855345	2.66E-06	25.60%
	2016	*_**SNP13363**_	A08	855373	2.66E-06	25.60%
	2016	*SNP13364	A08	855379	2.66E-06	25.60%
	2016	*SNP13365	A08	855462	4.45E-06	25.76%
	2016	*SNP13366	A08	855478	2.66E-06	25.60%

	2016	*_**SNP13464**_	A08	12628002	1.77E-05	27.68%
	2016	*SNP20416	B02	23772819	2.66E-06	25.60%
	2016	*_**SNP20417**_	B02	23772874	2.66E-06	25.60%
	2016	*SNP20418	B02	23772894	2.66E-06	25.60%
	2016	*SNP20419	B02	23772954	2.28E-06	27.55%
	2016	*_**SNP32483**_	B08	100913450	9.54E-06	28.49%
	2016	**SNP33973**	B09	61666165	1.61E-05	25.93%
	2017	*_**SNP02686**_	A02	46706823	7.00E-06	22.96%
	2017	**SNP19662**	B01	109604369	2.12E-05	17.64%
	2017	**SNP24480**	B04	46296118	1.63E-05	16.87%
	2017	SNP24481	B04	46296199	1.63E-05	16.87%
AFB_2	2015	**SNP27554**	B05	141021715	2.26E-05	22.27%
	2016	SNP00538	A01	34209806	2.72E-05	25.33%
	2016	_**SNP00539**_	A01	34209850	2.20E-05	25.90%
	2016	SNP00541	A01	34209885	2.59E-05	25.46%
	2016	SNP00542	A01	34209906	2.59E-05	25.46%
	2016	SNP00543	A01	34209910	2.59E-05	25.46%
	2016	_**SNP02428**_	A02	32402181	5.10E-06	31.15%
	2016	_**SNP05994**_	A04	5449452	1.96E-05	26.45%
	2016	SNP06001	A04	5449524	1.96E-05	26.45%
	2016	_**SNP06730**_	A04	41670901	1.54E-05	26.35%
	2016	SNP06731	A04	41670932	2.37E-05	25.52%
	2016	_**SNP07247**_	A04	69306120	3.23E-06	25.41%
	2016	_**SNP11095**_	A06	44574792	1.94E-05	25.92%
	2016	SNP11099	A06	44574866	1.94E-05	25.92%
	2016	_**SNP11310**_	A06	57503242	2.18E-05	26.66%
	2016	SNP13362	A08	855345	3.29E-06	25.10%
	2016	_**SNP13363**_	A08	855373	3.29E-06	25.10%
	2016	SNP13364	A08	855379	3.29E-06	25.10%
	2016	SNP13365	A08	855462	5.19E-06	25.16%
	2016	SNP13366	A08	855478	3.29E-06	25.10%
	2016	_**SNP13464**_	A08	12628002	2.24E-05	26.93%
	2016	SNP20416	B02	23772819	3.29E-06	25.10%
	2016	_**SNP20417**_	B02	23772874	3.29E-06	25.10%
	2016	SNP20418	B02	23772894	3.29E-06	25.10%
	2016	SNP20419	B02	23772954	2.84E-06	27.08%
	2016	_**SNP32483**_	B08	100913450	1.38E-05	27.35%
	2017	_**SNP02686**_	A02	46706823	4.85E-06	31.70%
	2017	**SNP19994**	B01	134968371	1.49E-05	28.91%

AFB1: aflatoxin B1 content; AFB2: aflatoxin B2 content; *: SNP marks significantly associated with both AFB1 and AFB2; Env: environment; PVE: The percentage of phenotypic variation explained; Peak SNP markers were highlighted in blod; Peak SNP markers associated with both AFB1 and AFB2 were highlighted in italic.

Table S3. Candidate genes information.

SNP Peak	Chromosome	Start Position	End Position	Name	ID	Gene Annotation
SNP27554	B05	141,029,915	141,040,113	Araip.524S2	158018	beta-amyrin synthase isoform X1 [Glycine max]
		140,916,101	140,931,206	Araip.P2YH6	160918	beta-amyrin synthase isoform X1 [Glycine max]
		140,965,278	140,978,530	Araip.Z78PS	160956	beta-amyrin synthase-like isoform 1 [Glycine max]
		141,108,722	141,116,901	Araip.675M1	159868	beta-amyrin synthase isoform X1 [Glycine max]
		141,118,279	141,119,333	Araip.L60XB	159919	beta-amyrin synthase-like isoform X2 [Glycine max]
SNP02428	A02	32,318,557	32,320,526	Aradu.L7CSQ	63440	cell cycle regulated microtubule associated protein
		32,326,522	32,327,036	Aradu.JY46G	63468	zinc finger MYM-type protein 1-like [Glycine max]
		32,327,059	32,328,823	Aradu.C0KGU	63454	zinc finger MYM-type protein 1-like [Glycine max]
		32,332,743	32,336,940	Aradu.87YLX	63428	putative protein TPRXL-like isoform X2 [Glycine max]
		32,418,675	32,419,185	Aradu.C7Q55	64658	Transposon protein
		32,432,180	32,433,008	Aradu.89PGL	64668	Transposon protein
		32,433,038	32,442,330	Aradu.SU4AL	64674	protein FAR-RED IMPAIRED RESPONSE 1-like [Glycine max]
		32,442,592	32,446,705	Aradu.GZ0CM	64645	Protein kinase superfamily protein
SNP05994	A04	5,372,133	5,376,424	Aradu.Y7IE8	197823	calcium-binding EF hand protein
		5,363,391	5,366,627	Aradu.IY1LP	197785	extracellular ligand-gated ion channel protein
		5,367,208	5,368,267	Aradu.N2LUB	197857	RPM1 interacting protein
		5,378,409	5,380,628	Aradu.15QSN	197797	short-chain dehydrogenase-reductase B
		5,431,058	5,433,961	Aradu.5BK9D	196631	serine threonine-protein phosphatase 7 long form homolog [Glycine max]
		5,464,319	5,467,605	Aradu.KZ75F	196645	acyl-protein thioesterase
		5,471,863	5,474,933	Aradu.7SV97	196592	mitochondrial pyruvate carrier 1-like isoform X3 [Glycine max]
		5,532,515	5,535,977	Aradu.6W9YP	188716	alkaline phytoceramidase
		5,538,767	5,543,953	Aradu.Q8U49	188753	Phosphatidate cytidylyltransferase family protein
		5,545,399	5,547,469	Aradu.R9QX8	188815	myosin-1-like isoform X3 [Glycine max]
SNP06730	A04	41,724,561	41,733,666	Aradu.EPT6Q	164196	sulfate transporter
		41,736,139	41,745,798	Aradu.LX8BH	164228	sulfate transporter

SNP0724 7	A04	69,218,571	69,229,314	Aradu.5T6BE	167610	1-aminocyclopropane-1-carboxylate oxidase homolog 1 [Glycine max]
		69,342,836	69,346,438	Aradu.HI72E	167906	F-box FRNI-like superfamily protein (Leucine-rich repeat)
		69,360,627	69,364,123	Aradu.E7174	168919	signal recognition particle receptor subunit alpha-like [Glycine max]
SNP1131 0	A06	57,409,126	57,414,188	Aradu.A3CPU	109972	dicer-like protein
		57,414,649	57,415,835	Aradu.MH3SJ	109998	protein FAR1-RELATED SEQUENCE 9-like isoform X5 [Glycine max]
		57,416,645	57,418,997	Aradu.CDM5G	109989	FAR1-related sequence
		57,446,064	57,452,035	Aradu.3VW4U	109952	protein FAR1-RELATED SEQUENCE 3-like isoform X1 [Glycine max]
		57,455,560	57,457,295	Aradu.Y38TJ	110037	B3 domain-containing transcription factor VRN1-like isoform X1 [Glycine max]
		57,473,760	57,475,704	Aradu.XRK81	110006	GRF zinc finger protein
		57,497,679	57,500,862	Aradu.79876	110527	probable glycosyltransferase At5g03795-like [Glycine max]
		57,501,750	57,506,178	Aradu.HU7TQ	110602	1-acyl-sn-glycerol-3-phosphate acyltransferase
		57,585,559	57,587,354	Aradu.X5E0S	110557	FAR1-RELATED SEQUENCE 3-like isoform X2 [Glycine max]
SNP1336 3	A08	761,144	762,370	Aradu.1HN9G	206497	CBS domain-containing protein CBSCBSPB1-like isoform X4 [Glycine max]
		762,799	763,959	Aradu.8L64B	206405	titin-like [Glycine max]
		765,745	766,609	Aradu.U2YMJ	206409	hypothetical protein
		775,128	777,415	Aradu.WD8GP	206435	isoflavone reductase-like protein-like [Glycine max]
		779,418	780,091	Aradu.FYE06	206489	uncharacterized protein LOC100809566 [Glycine max]
		781,121	782,168	Aradu.H02TL	206480	Rer1 family protein
		782,301	784,801	Aradu.D8UEG	206419	pfkB-like carbohydrate kinase family protein
		803,815	811,395	Aradu.F2JWB	243973	Glucose-1-phosphate adenylyltransferase family protein
		818,650	828,917	Aradu.7KG7Z	244013	DNA binding protein
		845,072	850,594	Aradu.J3YIL	243954	TATA-box-binding protein isoform X2 [Glycine max]
		883,361	883,990	Aradu.0Y260	243950	uncharacterized protein LOC102661892 [Glycine max]
		887,534	890,013	Aradu.S2G0W	237317	Cytochrome P450 superfamily protein
		894,303	899,403	Aradu.41JSL	237277	Inositol-pentakisphosphate 2-kinase family protein
		898,070	902,912	Aradu.694S8	237336	Sec14p-like phosphatidylinositol transfer family protein

		928,824	931,842	Aradu.N94TC	237366	Wound-responsive family protein
		952,988	956,174	Aradu.45U0D	237301	transcription factor bHLH123-like isoform X2 [Glycine max]
SNP13464	A08	12,543,004	12,545,369	Aradu.RM26Y	205579	Pollen Ole e 1 allergen and extensin family protein
		12,592,779	12,596,001	Aradu.QZ6DW	249116	phosphoglycerate Fbisphosphoglycerate mutase
		12,596,647	12,599,413	Aradu.SQ2UE	249168	Sec14p-like phosphatidylinositol transfer family protein
		12,600,594	12,611,186	Aradu.WYX50	249152	beta-xylosidase
		12,613,711	12,613,983	Aradu.2V3PB	249181	peroxisomal targeting signal type 2 receptor
		12,613,987	12,614,297	Aradu.UM0KS	249187	peroxisomal targeting signal type 2 receptor
		12,636,479	12,640,574	Aradu.00WD1	249096	Transducin FWD40 repeat-like superfamily protein
		12,655,378	12,658,568	Aradu.LCM6E	249138	ser Fthr-rich protein T10 in DGCR region-like protein
		12,725,780	12,728,015	Aradu.E735W	220281	trehalose-6-phosphate phosphatase
SNP20417	B02	23,691,982	23,694,742	Araip.V5FD4	184545	RNA-binding protein 38-like isoform X2 [Glycine max]
		23,811,435	23,818,809	Araip.BUD2P	184127	uncharacterized protein LOC100803479 isoform X3 [Glycine max]
SNP32483	A02	46,673,659	46,674,816	Aradu.W0PPM	66632	ATP-citrate lyase B-1
SNP19994	B01	135,001,287	135,004,376	Araip.S9R4E	409083	receptor-like protein kinase (Leucine-rich repeat)
		134,867,304	134,871,181	Araip.2S44I	405537	Cytochrome c oxidase subunit Vib family protein
		134,888,461	134,889,606	Araip.MS6UX	405533	protein kinase family protein
		134,937,237	134,941,981	Araip.V3WGE	408229	Reticulon family protein
		134,946,713	134,953,395	Araip.MC99C	408250	actin-binding FH2 (formin 2) family protein
		134,960,286	134,964,649	Araip.AML9J	408196	fatty acid desaturase 6
		134,965,272	134,966,780	Araip.L9ZIB	408222	BTB POZ domain-containing protein
		134,980,647	134,986,689	Araip.6FG7P	409092	ubiquitin carboxyl-terminal hydrolase
		135,023,735	135,027,751	Araip.X4GRJ	409043	WRKY family transcription factor family protein
		135,028,997	135,033,508	Araip.L90BS	409029	DNA glycosylase superfamily protein
		135,040,354	135,041,557	Araip.RF7PV	409118	serine threonine-protein phosphatase 7 long form homolog [Glycine max]
		135,046,386	135,050,634	Araip.W41VB	409057	Cytosol aminopeptidase family protein
		135,052,043	135,054,445	Araip.Z254D	409149	endonuclease exonuclease phosphatase family protein
		135,054,742	135,057,795	Araip.SCJ6B	409131	transmembrane 9 superfamily member 4-like [Glycine max]
		135,057,876	135,064,192	Araip.D5K21	409193	cyclic nucleotide-gated ion channel-like protein

		135,066,301	135,067,316	Araip.AZY85	409165	early nodulin-like protein
		135,068,030	135,071,797	Araip.W5GXX	409172	signal recognition particle receptor alpha subunit family protein
SNP05985	A04	5,372,133	5,376,424	Aradu.Y7IE8	197823	calcium-binding EF hand protein
		5,363,391	5,366,627	Aradu.IY1LP	197785	extracellular ligand-gated ion channel protein
		5,367,208	5,368,267	Aradu.N2LUB	197857	RPM1 interacting protein
		5,378,409	5,380,628	Aradu.15QSN	197797	short-chain dehydrogenase-reductase B
		5,380,015	5,384,204	Aradu.VS58R	197874	uncharacterized membrane protein At1g16860-like isoform X2 [Glycine max]
		5,431,058	5,433,961	Aradu.5BK9D	196631	serine threonine-protein phosphatase 7 long form homolog [Glycine max]
		5,464,319	5,467,605	Aradu.KZ75F	196645	acyl-protein thioesterase
		5,471,863	5,474,933	Aradu.7SV97	196592	mitochondrial pyruvate carrier 1-like isoform X3 [Glycine max]
		5,532,515	5,535,977	Aradu.6W9YP	188716	alkaline phytoceramidase
		5,538,767	5,543,953	Aradu.Q8U49	188753	Phosphatidate cytidylyltransferase family protein
		5,545,399	5,547,469	Aradu.R9QX8	188815	myosin-1-like isoform X3 [Glycine max]
SNP33973	B09	61,691,468	61,696,537	Araip.JAU44	247151	ubiquinone biosynthesis protein
SNP19662	B01	109,596,120	109,599,450	Araip.BP8BX	415866	lysosomal beta glucosidase-like isoform X2 [Glycine max]
		109,624,005	109,624,675	Araip.ECK9I	415855	integral membrane Yip1 family protein
		109,624,708	109,626,144	Araip.VKP29	415916	uncharacterized protein LOC100806270 isoform X1 [Glycine max]
		109,659,420	109,664,154	Araip.RE11A	415886	calcium-transporting ATPase
SNP24480	B04	46,330,823	46,331,238	Araip.83X32	435216	Protein disulfide isomerase (PDI)-like protein

Table S4. Pearson correlation for AFB1 and AFB2.

Traits	Pearson Correlation	*p*-Value
2015AFB$_1$ vs 2016AFB$_1$	0.37	<0.01
2015AFB$_1$ vs 2017AFB$_1$	0.29	<0.01
2016AFB$_1$ vs 2017AFB$_1$	0.28	<0.01
2015AFB$_2$ vs 2016AFB$_2$	0.41	<0.01
2015AFB$_2$ vs 2017AFB$_2$	0.17	0.08
2016AFB$_2$ vs 2017AFB$_2$	0.25	<0.05

Table S5. Meteorological data of peanut cultivation.

Year	Month	TC_air (°C)[a]	RH (%)[b]	Precipitation (mm)[c]	TC_soil (°C)[d]
	May	23.01 ± 3.22	72.95 ± 14.52	165.66	19.22 ± 1.14
	Jun	25.71 ± 3.57	76.65 ± 13.28	199.01	22.67 ± 1.26
2015	Jul	27.355 ± 3.61	75.02 ± 13.60	290.23	24.97 ± 0.82
	Aug	27.98 ± 3.57	71.34 ± 15.48	74.82	26.45 ± 0.54
	Sep	24.11 ± 3.30	73.34 ± 15.00	75.71	24.19 ± 1.15
	May	21.23 ± 3.87	74.39 ± 14.53	70.11	18.47 ± 0.62
	Jun	25.07 ± 4.04	76.97 ± 13.91	360.27	21.92 ± 1.11
2016	Jul	28.96 ± 3.82	77.35 ± 13.65	676.11	25.48 ± 1.44
	Aug	29.00 ± 3.96	75.28 ± 13.99	163.82	27.18 ± 1.00
	Sep	24.97 ± 4.07	68.76 ± 15.96	7.55	25.10 ± 0.82
	May	23.35 ± 4.48	67.36 ± 18.21	85.43	18.72 ± 1.20
	Jun	25.66 ± 3.06	75.92 ± 13.63	148.6	22.28 ± 0.83
2017	Jul	30.59 ± 3.73	70.31 ± 15.25	52.73	26.27 ± 1.78
	Aug	28.49 ± 3.44	79.78 ± 11.82	168.7	28.79 ± 0.66
	Sep	23.57 ± 3.05	82.97 ± 13.29	107.74	25.72 ± 0.78

a Average air temperature; b Average relative humidity; c monthly precipitation; d Average soil temperature.

Table S6. Soil nutrient content in the experimental field.

ENV [a]	Soil pH	SOM (%) [b]	STN (mg/kg) [c]	SAP (mg/kg) [d]	SAK (mg/kg) [e]
2015	7.80 ± 0.11	2.11 ± 0.03	77.72 ± 5.99	4.68 ± 0.77	137.25 ± 6.74
2016	7.77 ± 0.07	2.25 ± 0.15	90.88 ± 2.75	6.06 ± 1.71	125.71 ± 15.77
2017	8.01 ± 0.06	2.01 ± 0.31	87.68 ± 10.38	7.36 ± 2.01	116.88 ± 22.84

a environments; b soil organic matter content; c soil total nitrogen content; d soil available phosphorous content; e soil available potassium.

Table S7. Sequences of adapters used in ligation reaction.

Adapters	Sequences (5′–3′)
SacI01-T	ACACTCTTTCCCTACACGACGCTCTTCCGATCTGCATAAGCT
SacI01-B	p-TATGCAGATCGGAAGAGCGTCGTGTAGGGAAAGAGTGT
SacI02-T	ACACTCTTTCCCTACACGACGCTCTTCCGATCTGTACAAGCT
SacI02-B	p-TGTACAGATCGGAAGAGCGTCGTGTAGGGAAAGAGTGT
SacI03-T	ACACTCTTTCCCTACACGACGCTCTTCCGATCTACTGAAGCT
SacI03-B	p-TCAGTAGATCGGAAGAGCGTCGTGTAGGGAAAGAGTGT
SacI04-T	ACACTCTTTCCCTACACGACGCTCTTCCGATCTTACGAAGCT
SacI04-B	p-TCGTAAGATCGGAAGAGCGTCGTGTAGGGAAAGAGTGT
SacI05-T	ACACTCTTTCCCTACACGACGCTCTTCCGATCTCAGACAGCT
SacI05-B	p-GTCTGAGATCGGAAGAGCGTCGTGTAGGGAAAGAGTGT
SacI06-T	ACACTCTTTCCCTACACGACGCTCTTCCGATCTCGATCAGCT

SacI06-B	p-GATCGAGATCGGAAGAGCGTCGTGTAGGGAAAGAGTGT
SacI07-T	ACACTCTTTCCCTACACGACGCTCTTCCGATCTTTAGCAGCT
SacI07-B	p-GCTAAAGATCGGAAGAGCGTCGTGTAGGGAAAGAGTGT
SacI08-T	ACACTCTTTCCCTACACGACGCTCTTCCGATCTATCGCAGCT
SacI08-B	p-GCGATAGATCGGAAGAGCGTCGTGTAGGGAAAGAGTGT
SacI09-T	ACACTCTTTCCCTACACGACGCTCTTCCGATCTCGTATAGCT
SacI09-B	p-ATACGAGATCGGAAGAGCGTCGTGTAGGGAAAGAGTGT
SacI10-T	ACACTCTTTCCCTACACGACGCTCTTCCGATCTGCCATAGCT
SacI10-B	p-ATGGCAGATCGGAAGAGCGTCGTGTAGGGAAAGAGTGT
SacI11-T	ACACTCTTTCCCTACACGACGCTCTTCCGATCTAGACTAGCT
SacI11-B	p-AGTCTAGATCGGAAGAGCGTCGTGTAGGGAAAGAGTGT
SacI12-T	ACACTCTTTCCCTACACGACGCTCTTCCGATCTTAGCTAGCT
SacI12-B	p-AGCTAAGATCGGAAGAGCGTCGTGTAGGGAAAGAGTGT
SacI13-T	ACACTCTTTCCCTACACGACGCTCTTCCGATCTAGCTAAGCT
SacI13-B	p-TAGCTAGATCGGAAGAGCGTCGTGTAGGGAAAGAGTGT
SacI14-T	ACACTCTTTCCCTACACGACGCTCTTCCGATCTCAGTAAGC
SacI14-B	p-TACTGAGATCGGAAGAGCGTCGTGTAGGGAAAGAGTGT
SacI15-T	ACACTCTTTCCCTACACGACGCTCTTCCGATCTCGTCAAGCT
SacI15-B	p-TGACGAGATCGGAAGAGCGTCGTGTAGGGAAAGAGTGT
SacI16-T	ACACTCTTTCCCTACACGACGCTCTTCCGATCTATGCAAGCT
SacI16-B	p-TGCATAGATCGGAAGAGCGTCGTGTAGGGAAAGAGTGT
SacI17-T	ACACTCTTTCCCTACACGACGCTCTTCCGATCTGCTACAGCT
SacI17-B	p-GTAGCAGATCGGAAGAGCGTCGTGTAGGGAAAGAGTGT
SacI18-T	ACACTCTTTCCCTACACGACGCTCTTCCGATCTTGCACAGCT
SacI18-B	p-GTGCAAGATCGGAAGAGCGTCGTGTAGGGAAAGAGTGT
SacI19-T	ACACTCTTTCCCTACACGACGCTCTTCCGATCTACGTCAGCT
SacI19-B	p-GACGTAGATCGGAAGAGCGTCGTGTAGGGAAAGAGTGT
SacI20-T	ACACTCTTTCCCTACACGACGCTCTTCCGATCTAATGCAGCT
SacI20-B	p-GCATTAGATCGGAAGAGCGTCGTGTAGGGAAAGAGTGT
SacI21-T	ACACTCTTTCCCTACACGACGCTCTTCCGATCTCTGATAGCT
SacI21-B	p-ATCAGAGATCGGAAGAGCGTCGTGTAGGGAAAGAGTGT
SacI22-T	ACACTCTTTCCCTACACGACGCTCTTCCGATCTGACTTAGCT
SacI22-B	p-AAGTCAGATCGGAAGAGCGTCGTGTAGGGAAAGAGTGT
SacI23-T	ACACTCTTTCCCTACACGACGCTCTTCCGATCTGATCTAGCT
SacI23-B	p-AGATCAGATCGGAAGAGCGTCGTGTAGGGAAAGAGTGT
SacI24-T	ACACTCTTTCCCTACACGACGCTCTTCCGATCTTCAGTAGCT
SacI24-B	p-ACTGAAGATCGGAAGAGCGTCGTGTAGGGAAAGAGTGT
MseI01-T	p-TATATGCAGATCGGAAGAGCGGTTCAGCAGGAATGCCGAG
MseI01-B	CTCGGCATTCCTGCTGAACCGCTCTTCCGATCTGCATA
MseI02-T	p-TATGTACAGATCGGAAGAGCGGTTCAGCAGGAATGCCGAG
MseI02-B	CTCGGCATTCCTGCTGAACCGCTCTTCCGATCTGTACA
MseI03-T	p-TATCAGTAGATCGGAAGAGCGGTTCAGCAGGAATGCCGAG
MseI03-B	CTCGGCATTCCTGCTGAACCGCTCTTCCGATCTACTGA
MseI04-T	p-TATCGTAAGATCGGAAGAGCGGTTCAGCAGGAATGCCGAG
MseI04-B	CTCGGCATTCCTGCTGAACCGCTCTTCCGATCTTACGA
MseI05-T	p-TAGTCTGAGATCGGAAGAGCGGTTCAGCAGGAATGCCGAG
MseI05-B	CTCGGCATTCCTGCTGAACCGCTCTTCCGATCTCAGAC
MseI06-T	p-TAGATCGAGATCGGAAGAGCGGTTCAGCAGGAATGCCGAG
MseI06-B	CTCGGCATTCCTGCTGAACCGCTCTTCCGATCTCGATC
MseI07-T	p-TAGCTAAAGATCGGAAGAGCGGTTCAGCAGGAATGCCGAG
MseI07-B	CTCGGCATTCCTGCTGAACCGCTCTTCCGATCTTTAGC
MseI08-T	p-TAGCGATAGATCGGAAGAGCGGTTCAGCAGGAATGCCGAG

MseI08-B	CTCGGCATTCCTGCTGAACCGCTCTTCCGATCTATCGC
MseI09-T	p-TACTACGAGATCGGAAGAGCGGTTCAGCAGGAATGCCGAG
MseI09-B	CTCGGCATTCCTGCTGAACCGCTCTTCCGATCTCGTAG
MseI10-T	p-TACTGGCAGATCGGAAGAGCGGTTCAGCAGGAATGCCGAG
MseI10-B	CTCGGCATTCCTGCTGAACCGCTCTTCCGATCTGCCAG
MseI11-T	p-TACGTCTAGATCGGAAGAGCGGTTCAGCAGGAATGCCGAG
MseI11-B	CTCGGCATTCCTGCTGAACCGCTCTTCCGATCTAGACG
MseI12-T	p-TACGCTAAGATCGGAAGAGCGGTTCAGCAGGAATGCCGAG
MseI12-B	CTCGGCATTCCTGCTGAACCGCTCTTCCGATCTTAGCG
MseI13-T	p-TATAGCTAGATCGGAAGAGCGGTTCAGCAGGAATGCCGAG
MseI13-B	CTCGGCATTCCTGCTGAACCGCTCTTCCGATCTAGCTA
MseI14-T	p-TATACTGAGATCGGAAGAGCGGTTCAGCAGGAATGCCGAG
MseI14-B	CTCGGCATTCCTGCTGAACCGCTCTTCCGATCTCAGTA
MseI15-T	p-TATGACGAGATCGGAAGAGCGGTTCAGCAGGAATGCCGAG
MseI15-B	CTCGGCATTCCTGCTGAACCGCTCTTCCGATCTCGTCA
MseI16-T	p-TATGCATAGATCGGAAGAGCGGTTCAGCAGGAATGCCGAG
MseI16-B	CTCGGCATTCCTGCTGAACCGCTCTTCCGATCTATGCA
MseI17-T	p-TAGTAGCAGATCGGAAGAGCGGTTCAGCAGGAATGCCGAG
MseI17-B	CTCGGCATTCCTGCTGAACCGCTCTTCCGATCTGCTAC
MseI18-T	p-TAGTGCAAGATCGGAAGAGCGGTTCAGCAGGAATGCCGAG
MseI18-B	CTCGGCATTCCTGCTGAACCGCTCTTCCGATCTTGCAC
MseI19-T	p-TAGACGTAGATCGGAAGAGCGGTTCAGCAGGAATGCCGAG
MseI19-B	CTCGGCATTCCTGCTGAACCGCTCTTCCGATCTACGTC
MseI20-T	p-TAGCATTAGATCGGAAGAGCGGTTCAGCAGGAATGCCGAG
MseI20-B	CTCGGCATTCCTGCTGAACCGCTCTTCCGATCTAATGC
MseI21-T	p-TACTCAGAGATCGGAAGAGCGGTTCAGCAGGAATGCCGAG
MseI21-B	CTCGGCATTCCTGCTGAACCGCTCTTCCGATCTCTGAG
MseI22-T	p-TACAGTCAGATCGGAAGAGCGGTTCAGCAGGAATGCCGAG
MseI22-B	CTCGGCATTCCTGCTGAACCGCTCTTCCGATCTGACTG
MseI23-T	p-TACGATCAGATCGGAAGAGCGGTTCAGCAGGAATGCCGAG
MseI23-B	CTCGGCATTCCTGCTGAACCGCTCTTCCGATCTGATCG
MseI24-T	p-TACCTGAAGATCGGAAGAGCGGTTCAGCAGGAATGCCGAG
MseI24-B	CTCGGCATTCCTGCTGAACCGCTCTTCCGATCTTCAGG

The "p" at the end of sequence means that thenucleotide is 5′phosphorylated.

Table S8. Overhang primers for polymerase chain rection.

**Primer**	**Sequences (5′–3′)**
Forward	AATGATACGGCGACCACCGAGATCTACACTCTTTCCCTACACGACGCTCTTCCGATCT
Reverse	CAAGCAGAAGACGGCATACGAGATCGGTCTCGGCATTCCTGCTGAACCGCTCTTCCGATCT
